# Quality control stress test for deep learning-based diagnostic model in digital pathology

**DOI:** 10.1038/s41379-021-00859-x

**Published:** 2021-06-24

**Authors:** Birgid Schömig-Markiefka, Alexey Pryalukhin, Wolfgang Hulla, Andrey Bychkov, Junya Fukuoka, Anant Madabhushi, Viktor Achter, Lech Nieroda, Reinhard Büttner, Alexander Quaas, Yuri Tolkach

**Affiliations:** 1 Institute of Pathology, University Hospital Cologne, Cologne, Germany; 2 Institute of Pathology, Landesklinikum Wiener Neustadt, Wiener Neustadt, Austria; 3 Department of Pathology, Nagasaki University Graduate School of Biomedical Sciences, Nagasaki, Japan; 4 Department of Pathology, Kameda Medical Center, Kamogawa, Japan; 5 Department of Biomedical Engineering, Case Western Reserve University, Cleveland, OH USA; 6 Louis Stokes Cleveland Veterans Administration Medical Center, Cleveland, OH USA; 7 Regional computing center (RRZK), University of Cologne, Cologne, Germany

**Keywords:** Prostate cancer, Pathology

## Abstract

Digital pathology provides a possibility for computational analysis of histological slides and automatization of routine pathological tasks. Histological slides are very heterogeneous concerning staining, sections’ thickness, and artifacts arising during tissue processing, cutting, staining, and digitization. In this study, we digitally reproduce major types of artifacts. Using six datasets from four different institutions digitized by different scanner systems, we systematically explore artifacts’ influence on the accuracy of the pre-trained, validated, deep learning-based model for prostate cancer detection in histological slides. We provide evidence that any histological artifact dependent on severity can lead to a substantial loss in model performance. Strategies for the prevention of diagnostic model accuracy losses in the context of artifacts are warranted. Stress-testing of diagnostic models using synthetically generated artifacts might be an essential step during clinical validation of deep learning-based algorithms.

## Introduction

Digital pathology is an emerging transformation of diagnostic pathology [1]. At that, histological slides can be digitized using histological scanner and reviewed on the monitor without a microscope. One of the promises of digital pathology is an automated analysis of pathological specimens using deep learning-based models [2, 3]. Deep learning (DL) is a powerful technology for image analysis based on convolutional neural networks [3]. The latter use unsupervised feature generation by convolving images using multiple different filters and aggregate the corresponding representations in different ways to achieve a prediction. Many studies to date addressed the feasibility of DL for diagnostic applications with high accuracy [4–16] resulting in several commercial diagnostic products that are being developed for prostate cancer, breast cancer, skin diseases, and in other medical domains. However, this is still a work in progress.

Importantly, histological slides may be very heterogeneous with regard to staining, thickness of sections, and are especially prone to artifacts during tissue processing, cutting, staining, and digitization. Such heterogeneity is prominent among different institutions and even within the same laboratory. The aim of the pathologist and of any assisting system (e.g., DL-based) is to provide top diagnostic accuracy irrespective of pre-analytical issues and artifacts. Only few studies investigate how DL-based models will operate in case of artifacts [17–19]. Generalization of this information is difficult for models from different domains, tasks (classification, segmentation, detection), different neural network architectures, and models using different ground truth. However, this knowledge is of utmost importance for assisting systems to be implemented in the routine diagnostic workflow.

In this work we attempt to digitally reproduce major types of histological artifacts and perform a systematic stress test for a DL-based tool for prostate cancer (PCA) detection in histological slides which previously showed high accuracy (>98%) using external validation sets [4]. We investigate mechanism of misclassification in different types of artifacts and discuss strategies of how to prevent negative effects of artifacts on model accuracy during development and implementation of algorithm.

## Materials and methods

### Model description

The DL-based patch-level classification model for PCA detection used in the study was previously trained using a large-high-quality dataset based on the histological slides from The Cancer Genome Atlas (TCGA) PCA cohort containing ~1.5 million patches [4]. Several convolutional network architectures were tested with NASNetLarge and InceptionResNetV2 showing similar highest accuracy. In current implementation the model is based on the completely retrained InceptionResNetV2 architecture with fully connected layer containing 256 units and classification layer for three classes (prostate glandular tissue, nonglandular tissue, and tumor tissue). The patch size is 300 × 300 pixels (px) corresponding to the whole slide image (WSI) region size of ~150 × 150 µm at ×20 objective magnification (accounting for µm/px parameter of different scanning systems). For further details see ref. [4].

### Patient cases

Three independent patient cohorts and the original training cohort for control purposes were included in the study. Patients in all cohorts represented the full spectrum of PCA stages and Gleason grades from routine practice [4, 20]. Detailed characteristics of cohorts are outlined in Table 1.

**Table 1:** Characteristics of patient cohorts, datasets, and digitalization setup.

	Dataset 1^a^	Dataset 2	Dataset 3	Dataset 4	Dataset 5	Dataset 6
Source	TCGA	Case Western University	University Hospital Cologne	Hospital Wiener Neustadt		
Number of patients (prostate cancer)	362	15	81	51		
Number of slides	389	478	157	51	51	51
Slide scanner	Leica systems	Leica AT2	Hamamatsu S360	Leica GT450	Hamamatsu S360	Objective Imaging Glissando
Magnification	×40	×20	×40	×40	×40	×40
Scan resolution, µm/px	~0.25^b^	0.504	0.231	0.264	0.231	0.275
Number of patches (test dataset^c^): glandular nonglandular tumor	50,000 20,000 50,000	50,000 20,000 50,000	50,000 20,000 50,000	50,000 20,000 50,000	50,000 20,000 50,000	50,000 20,000 50,000
Test patch size	300 × 300 px (ca. 150 µm)					
Test magnification	×20					

^a^Dataset 1 was created from training dataset of the model implemented in this study.

^b^Some variations among different slides.

^c^Full extraction yielded large numbers of patches (up to ~7 million in Dataset 3); random selection from these full datasets with roughly similar number of patches from single slides was performed to construct test datasets due to high computational expenses of analysis of the full datasets.

### Digitization and datasets

Six datasets (DS1-6) were generated (Table 1). One cohort consisting of 51 patients (1 slide/patient) was digitized using three different scanners resulting in three datasets. For the final analysis, given very high computational costs, a random crop of each dataset with 120,000 patches was generated containing 50,000 patches with tumor tissue, 50,000 patches with nonneoplastic glandular prostate tissue, and 20,000 patches with nonglandular tissue (all properly classified by model at baseline, corresponding to reference F1 Score 1.0 for comparison with analysis in presence of artifacts). Generation of subsets was carried out randomly taking care for roughly similar number of patches per WSI for all three tissue classes.

### Artifacts

**Fig. 1:**
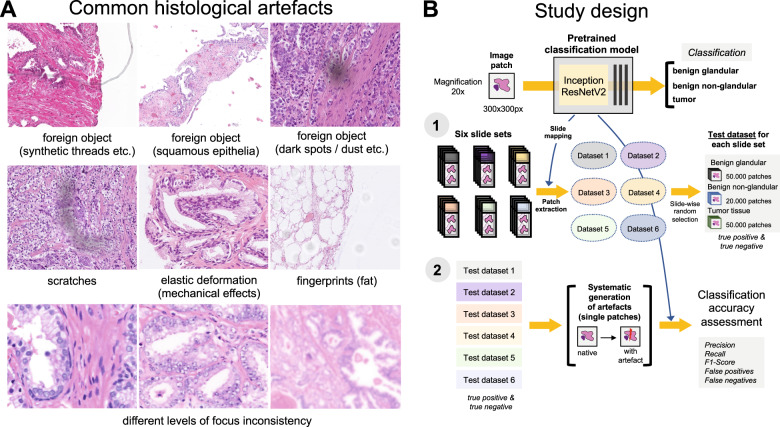
Common histological artifacts in digital pathology and study design. **A** Some of the common artifacts in digital pathology. **B** Study design. A pre-trained and validated model for prostate cancer detection [4] based on InceptionResNetV2 convolutional architecture was used in this study providing classification among benign glandular, benign nonglandular, and tumor tissue. Step 1: six test datasets were created (Supplementary Table 1 for characteristics) containing a random selection of only true positive (tumor class, 50,000 patches) and true negative patches (benign glandular class, 50,000 patches, and benign nonglandular class, 20,000 patches), correctly recognized by the model at baseline. Step 2: every of 12 studied artifacts or analytical situations were computationally generated for every single patch in six test datasets to test model accuracy in presence of these artifacts.

**Fig. 2:**
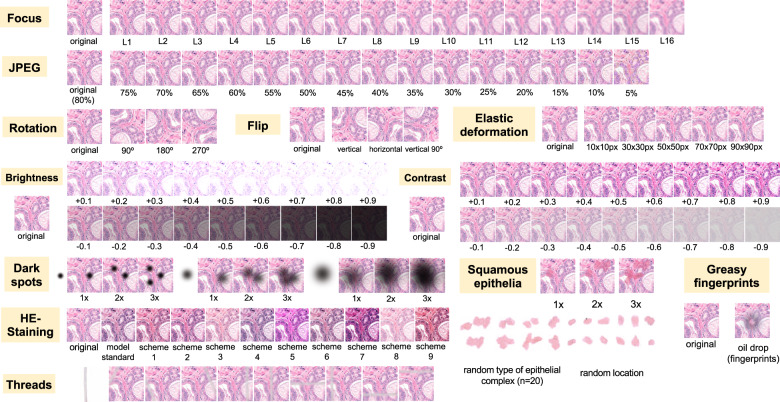
Twelve types of artifacts or analytical situations reproduced computationally in the study and their levels of severity. Flips and rotations are not typical artifacts, however, deep learning-based models were shown to be sensitive to context presentation in a patch, therefore these two analytical situations were included. Focus levels (L) represent consecutive increases of 2 px in kernel size of Gaussian filter with L1 corresponding to kernel size of 1 px. Grid size for elastic deformation is specified. Five levels were used with produced deformations visually staying in realistic range. Examples of synthetic thread, squamous epithelial cells, and fat vacuole resulting from fingerprints were extracted from routine whole slide images. Synthetic threads usually produce local focus deterioration and squamous cells, fingerprints, and dark spots usually do not affect focus. Representative images with staining schemes used for stain transfer in course of experiments as well as standard staining scheme used by model for stain normalization are presented in Supplementary Fig. 1.

Nine different types of artifacts common for routine histopathology practice were computationally generated. These artifacts included focus, elastic deformation, brightness, contrast, dark spots (e.g., dust, cover glass scratches, and other kinds of contamination), synthetic threads overlying tissue, contaminating squamous epithelia, greasy fingerprints on the slide surface, and hematoxylin-eosin (HE) staining scheme (Fig. 1). Some of the artefacts (focus, JPEG compression, elastic deformation, brightness, and contrast) were generated through corresponding changes of the image patch at a pixel level, while others (dark spots, synthetic threads, squamous epithelia, and fingerprints) were synthetized through overlaying of the corresponding artifact on the original pixel content, leaving the latter intact. In addition, three image parameters, potentially affecting performance of DL algorithm, such as jpeg compression, rotation of patch, and flipping of patch, were tested (Fig. 2). Three types of dark spots, ten variants of synthetic thread location, 20 variants of squamous epithelia, and a sample of oil drop (greasy fingerprints) were extracted from independent representative routine slides to be later overlaid on the single patches. Focus artifacts were generated using Gaussian blur with levels corresponding to 2 px increases in kernel size. Nine different staining schemes (Fig. 2, Supplementary Fig. 1) with different visual perception of staining quality (from poor to good) were saved as representative images from routine slides for stain transfer using Macenko algorithm [21]. Brightness normalization was routinely performed as the initial step of stain transfer. Representative images are provided in Fig. 2.

### Pipeline, software

The model was initially trained to work with stain normalization via Macenko stain transfer [21] from one standard staining scheme outlined in Supplementary Fig. 1 [4]. Therefore, stain-normalized versions of datasets were used in all analyses with exception of those used for HE-staining analysis (where nine other staining schemes were used for tests, Fig. 2, Supplementary Fig. 1). In a test pipeline, single artifacts were generated on every single patch of a corresponding dataset, and model classification was carried out on these modified patches to estimate model accuracy in presence of artifacts. Python (version 3.7.7) and Tensorflow (version 2.3) was used for model implementation. Staintools package was used for brightness standardization and Macenko stain transfer. Opencv-python and Pillow packages were used for image manipulations. Monai package (instrument: Rand2DElastic) was used for elastic deformations.

Principle component analysis and t-distributed stochastic neighbor embedding (t-SNE) modules from scikit-learn package for Python were used for analysis of similarity of image patch content. For this purpose, convolutional base of the model was disconnected from classification head and connected to three consecutive global average pooling layers to reduce the dimensions of output vector; output of the last convolutional layer with dimensions (8,8,1536) was therefore transformed to a vector (1,1,1536). Principal component analysis was performed using 700 components as first step with further t-SNE using 2 components and 3000 iterations. Visualization was performed using matplotlib package.

Gradient-weighted Class Activation Mapping (GradCAM) was implemented as described in [22] with outputs captured from activations of the last convolutional layer and opencv-python library for heatmap visualization.

### Hardware

Experiments were performed in parallel on two workstations with 2x GPU Nvidia RTX 2080 Ti 11 Gb and 1x GPU Quatro P6000 24 Gb, respectively, as well as using a high performing computing cluster of the University of Cologne equipped with 12 Nvidia V100 32 Gb GPU cards.

### Statistical analysis

Statistical analysis was performed using R version 4.0.3 (The R Foundation for Statistical Computing). Accuracy of the models was estimated using precision, recall, and F1 score metrics. Precision for tumor detection was calculated as TP/(TP + FP), recall as TP/(TP + FN) and F1 score as 2 × Precision × Recall/(Precision + Recall), where TP, FP, and FN are true positive, false positive and false negative patch classification results through model, respectively. Plots were created using ggplot2 and VennDiagram libraries for R.

## Results

### Dependence of model accuracy on the presence of artifacts

**Fig. 3:**
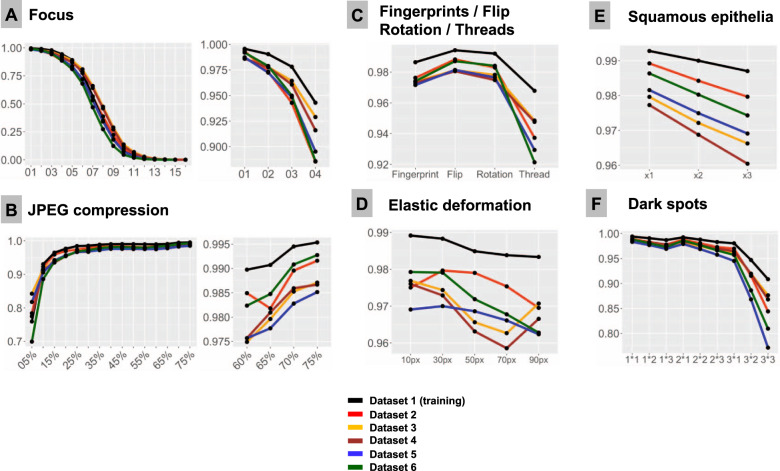
Systematic tests of model accuracy in six test datasets with computationally generated artifacts. F1-Score is used as accuracy metrics in all plots. All test dataset patches are properly classified by the model at baseline (baseline F1-Score is 1.0). Line colors correspond to test datasets (see legend). Note, that test dataset 1 stems from training dataset and serves as control measure. In all types of analysis, it demonstrates higher accuracy implying that model still remembers features inherent to this dataset. **A** Focus (*x*-axis represents levels of focus deterioration, corresponding to Fig. 2). **B** JPEG compression. **C** Four types of artifacts summarized in one plot: fat from fingerprints, flips, rotations of patch, and synthetic thread overlaying tissue. **D** Elastic deformations using different grid sizes. **E** Squamous epithelia: one, two, and three complexes (random selection of epithelial complex and location in patch; see Fig. 2) overlying tissue patch. **F** Dark spots: three types of dark spots with 1, 2, and 3 dark spots overlying tissue in patch at random location (e.g., 2 × 2 means that two dark spots of second type are overlaid on tissue in patch).

**Fig. 4:**
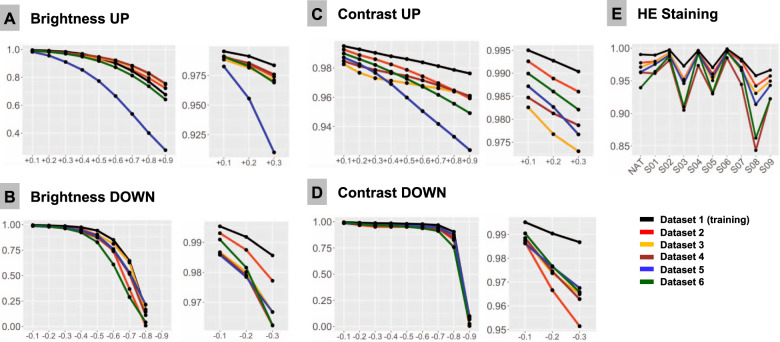
Systematic tests of model accuracy in six test datasets with computationally generated artifacts. F1-Score is used as accuracy metrics in all plots and is 1.0 at baseline for all patches in all datasets. Colors of lines correspond to test datasets (see legend). **A** Upregulated brightness. **B** Downregulated brightness. **C** Upregulated contrast. **D** Downregulated contrast. **E** Hematoxylin-eosin (HE) staining. NAT—native representation of the patch. S01-S09: stain transfer from one of the 9 staining schemes using Macenko algorithm (staining schemes presented Fig. 2, Supplementary Fig. 1).

**Fig. 5:**
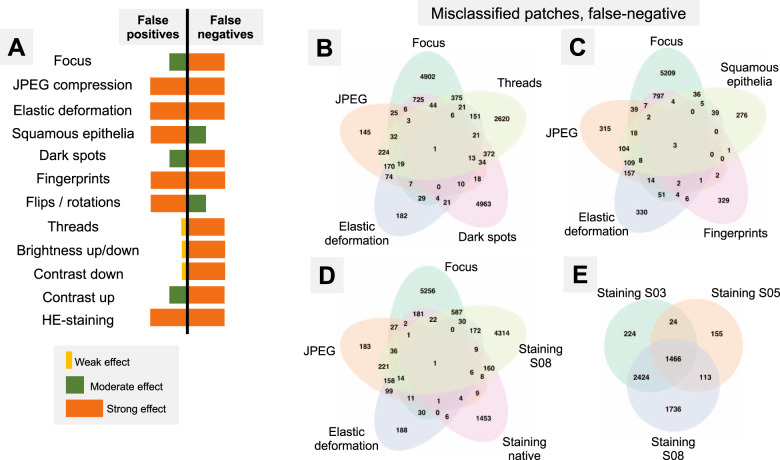
Analysis of artifact-induced misclassifications. **A** Summary of misclassification effects for twelve types of artifacts (all datasets) with regard to false positive (benign tissue classified as tumor) and false negative results (tumor tissue classified as benign); summary from Supplementary Figs. 2 and 3. Virtually all false-positive misclassification stem from benign glandular tissue with benign nonglandular tissue (fat, stroma, muscles, vessels, nerves, etc.) being resistant to misclassifications. **B**–**E** Venn diagrams of the misclassified patches (false-negative misclassifications of tumor tissue as benign) from Dataset 3. Following artifact severity levels were used for this representation: Focus level 4, JPEG compression 40%, elastic deformation with grid size 70 px, dark spot of third type with 2 spots overlying image patch, synthetic thread with random location, two random squamous epithelial cell complexes overlying image patch at random location, and native HE-staining scheme and stain transfer using S08 scheme (see Fig. 2). Venn diagrams demonstrate unique and intersecting misclassified patches among different types of artifacts. For false-positive misclassifications see Supplementary Fig. 4.

Six test datasets were used in this study, each containing 50,000 patches from tumor, 50,000 patches from benign glandular, and 20,000 patches from benign nonglandular prostate tissue classes (fat, stroma, muscle, vessels, etc.), all properly classified by model at baseline. Of those, Dataset 1 (details in Table 1) is composed of training dataset patches and included for control purposes. All studied artifacts reduced the accuracy of the pre-trained deep learning model for PCA detection [4] used in this study (Figs. 3, 4, and 5).

### Focus

Sixteen focus levels were tested corresponding to increments of 2 pixels in Gaussian blur kernel filter (Fig. 2). Even minimal focus deteriorations led to negative changes in model accuracy. Thus, with kernel sizes 1 px (level 1) and 3 px (level 2), still visually percepted as tolerable by pathologists, the number of false negative (FN) tumor patches was 372/50,000 (0.7%) and 2476/50,000 (4.95%), respectively, with progressive fall in accuracy thereafter (Fig. 3).

### JPEG compression

JPEG compression of 80% or equivalent is used by many scanner systems as a standard; therefore we used 80% as a baseline reference (F1 score = 1.0) and tested accuracy of the model at a range of compressions (Fig. 3B). We observed a slight fall in accuracy starting from 75% JPEG compression. However, rather high levels of accuracy (F1 score >0.95) were evident down to 15% compression level with sudden fall thereafter.

### Flip and rotation

Deep learning-based models are known to be sensitive to presentation of information in the image patch. Although not a typical histological artifact, we aimed to test the model classification consistency in case of simple flips and rotations in random directions. These led to minor losses of accuracy compared to baseline, with F1 score >0.98 for flips and >0.976 for random rotations in tested datasets (Fig. 3C).

### Elastic deformation

Elastic deformation of cells and tissue fibers has a mechanical distortion in its nature. It is a common artifact resulting from tissue cutting and tissue harvesting. We imitated elastic deformations using five sizes of grid cell staying in the visually realistic range of deformation. All types of deformation produced losses of accuracy with resulting F1 score ranging 0.959–0.980 and larger grid cell sizes associated with higher losses (Fig. 3D).

### Contrast and brightness

Different scanner systems provide a very different quality of the resulting images with regard to color scheme, brightness and contrast. Analysis of patch content similarity shows scanner-related clustering even when the same slides are scanned by different systems (Supplementary Fig. 5A). Negative and positive ranges of brightness and contrast were tested, with increments/decrements of 0.1 (10%), respectively (Fig. 4A–D). Notably, even small changes in contrast and brightness produced losses in accuracy which became more prominent by further relaxation. Upregulation of brightness and contrast had prominent effects in Dataset 5. Further investigation of this unexpected finding revealed that the WSI scanner was calibrated by the operator according to own visual perception of good contrast and brightness of scanned slides. All other datasets were scanned using calibrated manufacturer setup.

### Dark spots

Dark spots are probably the most common artifacts seen in digital pathology. They usually result from dust, scratches, and foreign objects on the surface of the histological slides or under the coverslip. These dark spots are often transparent and usually do not alter the focus. We reproduced 3 types of dark spots (Fig. 2). Placement of these artifacts on the tested images was generated randomly. For every type of spot one, two, and three spots overlying original patch were tested. Number of spots was positively correlated with losses of accuracy (Fig. 3F). Most prominent losses were evident for spot type 3 (the biggest one), especially with several such spots overlying single patches at the same time reducing transparency.

### Threads

Elongated subjects like synthetic threads are occasionally seen in digitized histological slides, usually accompanied by focal focus distortion. We reproduced 10 different positions of a thread/focus distortion with random selection of the position for single patches during tests (Fig. 2). This produced severe accuracy losses with F1 score dropped down to 0.92 from the baseline F1 score of 1.0 (Fig. 3C).

### Squamous epithelia

Squamous epithelia are one of the commonest artifacts resulting from contamination of the prostate biopsy specimen during tissue processing. They lie on the tissue’s surface, are to large extent transparent, and usually do not impair focus quality during scanning (Fig. 1). We extracted 20 templates of squamous epithelia consisting of one or more epithelial cells (Fig. 2). For each patch, one, two, or three random epithelial templates were overlaid before classification with position selected randomly. Accuracy loss was correlated with the number of overlaid epithelial complexes (Fig. 3E). We found that even a single squamous cell cluster produced small but non-negligible losses in accuracy in all datasets.

### Fingerprints (oil drops)

Fingerprints are common and appear as small transparent fat drops in digitized histological slides. We extracted a representative fat drop from WSI and overlaid it on original patches during experiments to see how it can affect the classification (Fig. 2). The classification accuracy was still high due to transparency of fat drops, however, non-negligible losses were evident (F1 score <0.98; Fig. 3C).

### Staining

HE-staining heterogeneity or just a visually “bad” staining (too intensive or too weak) is a prominent source of inter- and intra-institutional heterogeneity in histological slides. Our PCA detection model was developed to work with brightness standardization and stain normalization using a reference staining scheme and Macenko algorithm [21] (Supplementary Fig. 1). Baseline F1 score using this scheme is 1.0 in all datasets. We carried out model accuracy tests of native staining (without any normalization) as well as using a stain transfer from 9 visually different staining schemes including strong and weak staining for every single patch from test datasets (Fig. 2, Supplementary Fig. 1). Substantial losses of accuracy were observed in case of staining scheme visually interpreted as “poor” (Fig. 4E).

Additional level of color heterogeneity originates from the fact that every scanning system has a special color “touch” affecting original staining (Supplementary Fig. 5A). The similarity analysis of image patch content shows that even after Macenko stain transfer the clustering of image patches from different datasets is defined by the scanner system used (Supplementary Fig. 5B), even if this type of stain normalization allows for high model classification accuracy. Only style/stain transfer using generative adversarial network (GAN) (Supplementary Methods; Supplementary Fig. 5C) is able to override scanner-specific features and allow similarity clustering of single patches based on the histological content.

### Artifacts and tissue type

The artifacts affect the model accuracy through false positive (FP) or false negative (FN) results. Thus, nonglandular tissue (stroma, fat, muscle, nerves, vessels, etc.) is a nonsignificant source of FP patches, i.e., benign classified as tumor. The greatest number of FP patches in this tissue class was 138/20,000 (0.69%) in Dataset 6 at the 5% JPEG compression level; while in all other scenarios it was <0.08%. Therefore, misclassification occurs mostly in tumor (FN) and benign glandular (FP) classes which were, however, affected in different ways (Fig. 5A, detailed information in Supplementary Figs. 2 and 3). JPEG compression and fingerprints resulted in the similar numbers of FP and FN. Elastic deformation and HE staining produced both types of misclassification, however, predilection to FP and FN was depending on grid size and staining scheme used, respectively. Other artifacts also showed some propensity to certain types of misclassifications (Fig. 5A).

### Dataset-related heterogeneity

Dataset 1 composed of patches from training dataset provided better accuracy in presence of all artifacts, implying that the model still remembers some features inherent for this particular dataset. The trends of misclassification were in general similar for all datasets.

Importantly, during flips, rotations, elastic deformation, squamous epithelia, and upregulated contrast artifacts, Datasets 3–5 belonging to the same cohort, but scanned by different systems, showed slightly larger accuracy losses compared to Datasets 2 and 6. Deeper analysis of this phenomenon revealed that Datasets 3–5 showed up to 4 times more false-positive misclassifications compared to Datasets 2 and 6 with approximately the same number of false negative patches. Morphological analysis provides some clues and allows to attribute this to the fact that all benign patches in Datasets 3–5 were from regions immediately adjacent to tumor areas often carrying preneoplastic lesions (high-grade prostatic intraepithelial neoplasia, HGPIN) which contain cytological features of tumor tissue and, apparently, have lower threshold for misclassification. Benign class patches in Dataset 6 and, especially, dataset 2 stemmed to a large extent from completely “benign” slides or slides with a small amount of tumor. This is supported by the fact that baseline probability of being a tumor in glandular tissue class was statistically significant different between Datasets (DS) 2/6 and DS 3–5 (mean probability of being tumor for DS1 0.019, DS3 0.04, DS4 0.045, DS5 0.032, DS6 0.023; for any comparison: paired *t*-test < 1e−10). Moreover, F1 accuracy curves for single artifacts were often visually stratified by this baseline probability of being a tumor for benign patches.

### Misclassified patches

**Fig. 6:**
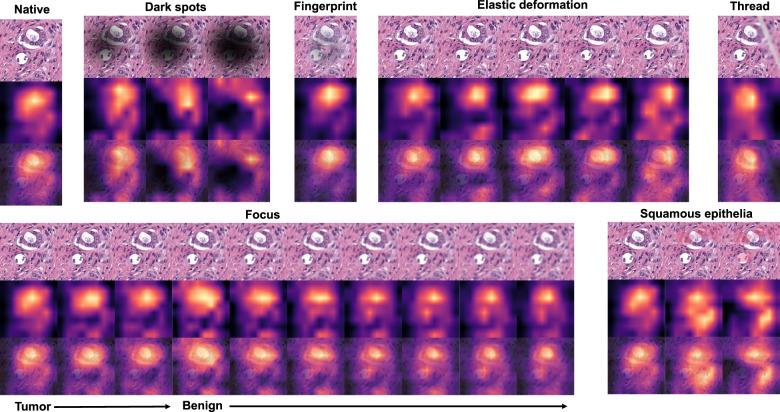
Gradient-weighted class activation mapping (GradCAM) analysis for different types of artifacts with model “attention” heatmaps (yellow and red representing areas in patch used by model for classification): one representative patch with from a tumor class (Dataset 3) is shown. Dark spots, fingerprints, synthetic threads and squamous epithelia in general do not generate new features and act by obfuscating patch content and existing features. During focus deterioration, even after misclassification (starting from focus level 4) to false negative benign category, roughly same regions are used for classification. On contrary, elastic deformations produce visible feature instability and volatility of patch regions used for classification implying potential of elastic deformations to generate new features.

**Fig. 7:**
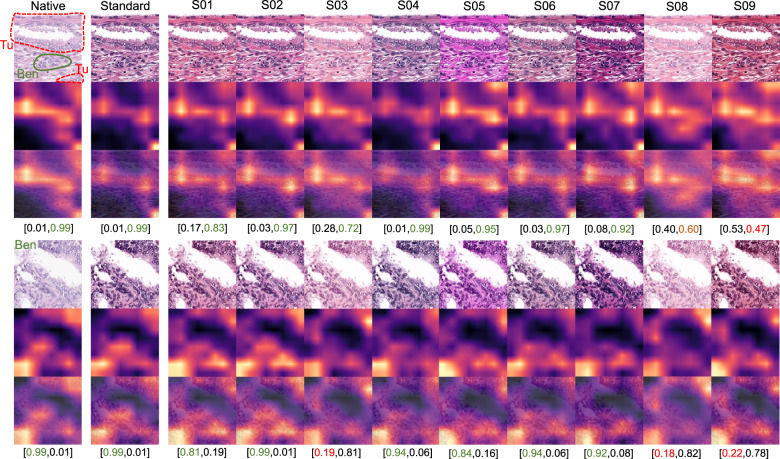
Gradient-weighted class activation mapping (GradCAM) analysis for hematoxylin-eosin staining as artifact. GradCAM “attention” heatmaps (yellow and red representing areas in patch used by model for classification) are presented: two representative patches from the tumor class (containing small benign gland as well) (above) and benign glandular class (below) are presented (Dataset 3). Analogous to elastic deformation (Fig. 6) different staining schemes seem to activate/deactivate different features (volatility of GradCAM heatmaps) which affect classification in these two patches. Model probability for classes [benign, tumor] is presented under every patch. Green color of font corresponds to proper classification, red—to misclassification. S01-S09 correspond to different staining schemes used for stain transfer in this study (Fig. 2, Supplementary Fig. 1).

As all studied datasets demonstrated the same patterns of model accuracy behavior in presence of artifacts, we selected a representative dataset (Dataset 3) for detailed investigation of misclassified patches. In this dataset intersections were evident among misclassified patches for different artifacts (Fig. 5B–E). The artifacts that act randomly and generate principally new content in patch (squamous epithelial cell, threads, dark spots) or substantially reduce the amount of existing information (focus, threads, dark spots) tend to show larger numbers of unique misclassified patches (Fig. 5B–E). GradCAM-powered analysis of class activation maps showed, however, that this new content is not being used for classification (new features) but rather obfuscating the existing features (Fig. 6). However, some artifacts seem to genuinely change the feature constellation or feature prominence in a patch (elastic deformation, HE-staining; Figs. 6 and 7). This is supported by the fact that elastic deformations produce the highest numbers of false positive results (Supplementary Fig. 4).

**Fig. 8:**
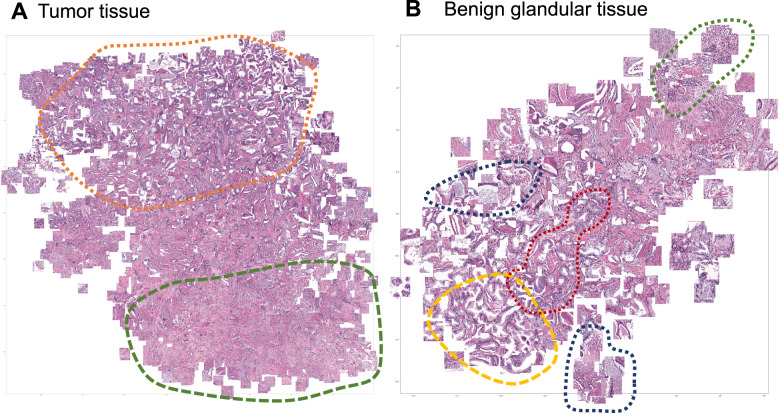
Similarity analysis for misclassified patches resulting from focus quality deterioration up to a level 4 (kernel size of 7 px for Gaussian blur filter). **A** False negative misclassifications of tumor tissue as benign (Dataset 3). **B** False-positive misclassifications of benign glandular tissue as tumor (Dataset 3). Several morphological clusters are evident for false negative results (**A**): orange—patches corresponding to well differentiated tumors with Gleason score 3 + 3 = 6 as well as tumors with pseudohyperplastic features, both naturally very similar to benign tissue; green—tumor patches with very low tumor content of few tumor glands or less. Four main morphological clusters can be identified for false positive results (**B**): dark blue—luminal content more typical for tumor tissue (necrotic debris, coarse eosinophilic material), yellow—retraction artifacts more typical for tumor tissue, red—preneoplastic changes (high-grade prostatic intraepithelial neoplasia), green—benign patches with inflammation infiltrate.

Focus artifacts are probably the most important artifacts in digital pathology. We carried out similarity analysis using our model as encoder (see Methods) for focus quality-related misclassified patches. The latter were clustered using t-SNE principle and analyzed by the expert genitourinary pathologist (YT) to identify the morphological descriptive features. False negative tumor patches built following recognizable clusters (Fig. 8A): (1) very low tumor content in patch (single gland or less), (2) well differentiated tumor or carcinoma with pseudohyperplastic features. Among false positive classified benign patches several clusters were evident (Fig. 8B): (1) inflammation, (2) luminal content of glands, (3) preneoplastic changes (HGPIN), and (4) retraction artifacts (commonly seen in tumor tissue).

## Discussion

Emerging digital transformation of diagnostic pathology provides a possibility for automatization of some pathological tasks [1]. Histological slides, digitized with high resolution, can be effectively analyzed using different computer vision algorithms [1, 2]. DL-based algorithms are especially accurate in this setting and were successfully implemented for tumor recognition, tumor grading, and extracting prognostic and predictive information from histological slides of patients with different types of cancer [4, 5, 7, 9–16, 23, 24]. In the real world setting histological slides are extremely heterogeneous even within single pathological laboratory and often contain multiple artifacts which can stem from natural reasons (mechanical or cauterization artifacts related to tissue sampling), preprocessing (embedding, fixation, cutting, staining) and digitization steps (focus, foreign objects, color scheme/brightness/contrast, level of image compression etc.) (Fig. 1). Most DL algorithms published to date did not explicitly control for effects of artifacts and, therefore, might be biased through the initial selection of higher quality slides for training and testing phases. High accuracy of DL-based algorithms in the presence of artifacts is of utmost importance for their implementation in the clinical workflow.

In this study, we employed a recently published DL-based model for PCA detection in histological slides developed on large-high-quality training dataset and validated on two external datasets with high overall accuracy of >98% [4]. We reproduced 12 types of common histological artifacts or situations (Fig. 2) and systematically tested how model accuracy may depend on their presence and severity using 6 datasets representing different institutes and different scanning systems (Table 1). As it was anticipated, all tested artifacts indeed affected model accuracy as they reduced the amount of information or distorted the information within an image (Figs. 3 and 4). Noteworthy, glandular tissue classes (tumor and benign) were the main source of artifacts, while nonglandular tissue contributed much less significantly.

Two main misclassification mechanisms (false positive tumor classification for benign tissue and false negative benign classification for tumor regions) differed among tested artifacts (Fig. 5) with a slight predominance of false negatives, which are naturally worse and can have poor diagnostic consequences as model fails silently. False-positive misclassifications will be normally highlighted by model and reviewed/controlled by pathologist operator.

Several published studies systematically addressed the influence of histological artifacts on performance of DL-based models in diagnostic pathology. A solid basis of evidence is related to focus problem in digital pathology [17, 25–28]. Most of the studies, however, concentrate on quantitative detection of out-of-focus regions using simple DL-based models trained on synthetic data [17, 25–28] or other principles [29, 30]. Kohlberger et al. [25] investigated accuracy losses of breast cancer metastasis detection model based on publicly available dataset and produced conclusions concordant with the results of our study. The advantage of our study is that we employed a validated model with high accuracy and were able to test it using 6 different datasets digitized with different WSI scanners. Wrong focus is an artifact with a clear mechanism of action through degradation of image features primarily associated with false-negative misclassifications of tumor regions which are dangerous as model fails silently (Fig. 5, Supplementary Fig. 5). Therefore, major digital pathology guidelines (The Royal College of Pathologists [31], College of American Pathologists [32]) require no objective threshold for focus quality, and pathologists decide whether the image sharpness is sufficient for unequivocal diagnosis. Our results show that substantial losses of accuracy can occur when the focus quality levels are still visually perceptible as adequate by pathologists (Fig. 3). We also provide morphological classification of image regions most susceptible to misclassification in case of focus artifacts (Fig. 7).

Image compression effects were investigated by several groups showing accuracy deterioration of models for metastatic cancer detection and segmentation, nuclei segmentation, and lymphocyte detection [17, 19]. Image compression is a fully controllable parameter set up manually during scanning (usually at a level of 80% or more). It normally does not undergo any changes after complete and successful validation of digital pathology system. However, lower levels of JPEG compression can be manually set up by WSI operator to reduce needs for a storage space or in research setting. Interestingly, the human eye does not recognize image alteration within a wide range of compression levels (30–90%). The results of the current study and other published studies [17, 33] show that this might have potential consequences for accuracy of analytical models trained on datasets with lower compression levels, should they be applied to such images. Our findings show that any compression levels under 80% can result in accuracy deterioration and should be avoided.

Hematoxylin-eosin staining is a factor which is extremely difficult (or even impossible) to standardize at pre-analytical stage. WSIs from the single laboratory and, especially, from different institutions have very different HE-staining schemes due to multitude of factors, such as variation of stains, reagents and manufacturers, protocols, room conditions, and many more [34] (Supplementary Fig. 1). Additional layer of heterogeneity are color schemes of WSI scanners from different manufactures which imprint every scanned slide [35, 36]. The same slides from one institution digitized by three different scanners (Datasets 3–5) in our study are highly heterogenous in terms of color scheme, brightness and contrast (Supplementary Fig. 4A), corroborating the results of the recent study by Schmitt et al. [34]. Obviously, staining is a parameter that should be controlled for at time of WSI analysis by diagnostic models. Substantial losses in accuracy dependent on HE-staining quality, brightness, and contrast were evident in our study (Fig. 4). Many methods were suggested for stain and color normalization with some of the most popular being Macenko [21], Vahadane [37], Bejnordi [38], and sparse autoencoder-based algorithms [39]. Also, DL-based pix2pix GANs have been extensively studied most recently providing not only stain normalization, but also style transfer [40–42]. Style transfer using GANs might be a promising approach to tailor model performance to single institutions where it should be implemented. In comparison to stain vector transfer using Macenko principle, pix2pix GANs can allow for a more advanced adaptation to the target domain and elimination of scanner-specific features [35] (see comparison in Supplementary Fig. 4B, C). Other approaches to address this problem are using extensive augmentation of training dataset via color/staining changes [18, 43, 44], using staining invariant features for model training [42, 45, 46], and using large training datasets from different institutions to compensate for the necessity of augmentation [13].

To the best of our knowledge, other artifacts investigated in our study (dark spots, overlying squamous epithelia, synthetic threads, elastic deformations, fingerprints) were not addressed systematically in the pathology domain and, therefore, represent the novelty of this study. All these artifacts might result in accuracy deterioration and warrant quality control measures.

Several strategies might be implemented to sustain accuracy of DL-based models in context of artifacts. Firstly, implementation of pre-analytical quality control tools (e.g. HistoQC [47]) is of utmost importance before models are even being applied to WSIs. Secondly, “knowing own models” principle is important to predict in which situations the former can fail and to achieve a balance between additional QC-related computational load and inference speed. Next, augmentation of training dataset using generation of synthetic artifacts can be a reasonable strategy to improve model accuracy. In addition, uncertainty measurements for a model classification might help to identify visual contexts to which the model was not exposed during training, such as artifacts [24]. And lastly, more standardization is warranted for digital pathology systems, particularly in terms of color calibration and image compression levels [48].

This study had certain limitations that require further elaboration. Although our model was trained using high-quality dataset, it is used only for detection of PCA. Generalizability of the results to other tumor types and DL models warrant additional investigations. We used the lowest levels of JPEG compression of 80% as reference. Some authors show that even lower compression levels (>90%) might be necessary for higher accuracy [17], although, we believe that it is a matter of compression level of images in training dataset (80% in our case). We do not reproduce certain types of artifacts like tissue folds (because of the technical difficulty of generation) and pen marks as the latter are basically not present in diagnostic setting (slides are being scanned before pathologists receive them) and can be easily avoided through slide cleaning before scanning in other situations. While our results are important for DL-based classification algorithms, it is not clear what impact the artifacts will have on non-DL-based computer vision algorithms (not addressed in this work).

We believe that our stress-testing pipeline might be a necessary step for any diagnostic model/algorithm in course of clinical validation. While our study was performed entirely on HE-stained slides, we may predict that histological artifacts play the similar detrimental role in more advanced staining techniques, such as histochemistry, immunohistochemistry, and immunofluorescence, particularly multiplexed. Immunostaining and immunofluorescence are widely utilized as objects for DL in pathology and impact of tissue artifacts should be investigated in future studies.

In our study, we reproduced twelve types of common histological artifacts or analytical situations. We systematically tested the effects of these artifacts and revealed mechanisms of misclassification using 6 datasets from several institutions, digitized by different scanning systems, using a validated pre-trained model for PCA detection with high accuracy. We here provide evidence that any histological artifact can lead to substantial loss of accuracy in DL model performance. We discuss the strategies for prevention of model accuracy losses in context of the artifacts. Stress-testing of diagnostic models using synthetically generated artifacts might be an important step during clinical validation of DL algorithms.

## Supplementary information

Supplementary Data

## Data Availability

Six generated datasets are available for download and academic usage at Zenodo (http://zenodo.org), Deposits: 4789576 (Dataset 1–4) and 4904569 (Datasets 5–6). The code used for artifact generation and dataset processing with model predictions is available for download at https://github.com/cpath-ukk/Artifact. The original whole slide images are available from corresponding authors on request.
