# Supplementary material for: Quality control stress test for deep learning-based diagnostic model in digital pathology
Source: Mod Pathol. 2021 Jun 24;34(12):2098–108. doi: 10.1038/s41379-021-00859-x (PMC8592835; doi:10.1038/s41379-021-00859-x)
Supplement: Supplementary file 1 — Supplementary Data [file 41379_2021_859_MOESM1_ESM.pdf]

Supplementary Data

"Quality control stress test for deep learning-based diagnostic model in digital pathology"

Schömig-Markiefka B. et al.

Supplementary Methods

Implementation of Pix2Pix generative adversarial network for stain normalization

Classical U-Net encoder-decoder generator model architecture (Ronneberger et al. 2015) was used for implementation of generative adversarial network with patch size 512x512 px (scaled from patches with absolute size of 150 µm scanned at 40x corrected for µm per pixel value of scanning system). Five thousand patches were randomly selected from tumor and gland-containing benign classes (Dataset 1) in their native version, which were split (3500:1500 patches) between training:test subsets controlling for non-intersection of subsets on the case level. A second dataset (=target domain) was generated from the same images with brightness standardization and stain transfer using standard scheme of the model (Suppl. Fig. 1) and Macenko algorithm. Pix2pix principle (Isola et al. 2016) for conditional adversarial network was implemented using Python (version 3.7.7) and Tensorflow (version 2.3). The model was trained with following parameters: batch size n image = 1, and the Adam version of stochastic gradient descent, for 100 epochs. Test results were evaluated visually and the weights of visually best performing model (captured at Epoch 35) were used for experiments.

References

Ronneberger O., Fischer P., Brox T. U-Net: Convolutional Networks for Biomedical Image Segmentation. arXiv:1505.04597.

Isola P, Zhu J.-Y., Zhou T., Efros A.A. Image-to-Image Translation with Conditional Adversarial Networks. arXiv:1611.07004

Supplementary Figures

Suppl. Fig. 1

Ten different hematoxylin-eosin staining schemes used for stain transfer in the study: one reference scheme from original publication used for color normalization during model implementation and nine schemes selected for experiments from routine slides (S01-S09) with different visual estimations of staining quality from "poor" to "good".

Suppl. Fig. 2

False positive (red circles) and false negative (black circles) misclassifications for focus (Levels 1-5), JPEG compression (5-75%), elastic deformation using grid sizes 10, 30, 50, 70, and 90px, squamous epithelia (1, 2, and 3 random overlying epithelial complexes at random location), dark spots of three types with 1, 2, and 3 darkspots of the same type overlying tissue in patch (e.g., 2x3 means dark spot of second type, shown in Figure 2, with three dark spots of this type simultaneously overlying a patch at random locations), fingerprints, flips, rotations, and synthetic thread with random location. Circles correspond to median number of misclassified patches across 6 test datasets. For reference purposes see Table below for translation of number of patches into % rate of false positive (FP) and false negative (FN) results.

Suppl. Fig. 3

False positive (red circles) and false negative (black circles) misclassifications for upregulated/downregulated brightness and contrast, and native HE-staining scheme as well as HE-staining schemes received through stain transfer (S01-S09 test schemes, see **Figure 2, Suppl. Fig. 1**). Circles correspond to median number of misclassified patches across 6 test datasets. For reference purposes see Table below for translation of number of patches into % rate of false positive (FP) and false negative (FN) results.

Suppl. Figure 4

Venn diagrams of the misclassified patches (false-positive misclassifications of benign tissue as tumor) from Dataset 3. Following artifact severity levels were used for this representation: Focus level 4, JPEG compression 40%, elastic deformation with grid size 70 px, dark spot of third type with 2 spots overlying image patch, synthetic thread with random location, two random squamous epithelial cell complexes overlying image patch at random location, and native HE-staining scheme and stain transfer using S08 scheme (see **Figure 2**).

Suppl. Fig. 5

t-distributed stochastic neighbor embedding (t-SNE) for embedding vectors of representative patches from tumor classes of Datasets 2, 3, 4, and 5 (random selection of 2000 patches / dataset), which were scanned by Leica AT2, Hamamatsu S360, Leica GT450, and Glissando Objective Imaging scanner, respectively (native color scheme without stain normalization) (**A**). Clustering patterns demonstrate prominent differences in color schemes. At that, Datasets 3, 4, and 5 represent the same physical histological slides scanned by different scanning systems. **B.** Same patches after Macenko stain transfer normalization using standard model staining scheme (**Figure 2, Suppl. Fig. 1**). Although visually patches appear to have similar color / staining scheme, they are still clustered together based on scanner system used. **C.** Pix2pix generative adversarial network (GAN) was trained (**Suppl. Methods**) for style transfer and stain normalization (using training dataset in native form as a source domain and stain normalized training dataset patches as a target domain; standard model staining scheme from original publication was used as reference for normalization). By similar visual appearance as in **(B)** GAN-based style transfer

eliminates scanning system-related features allowing clustering based on the morphological content.

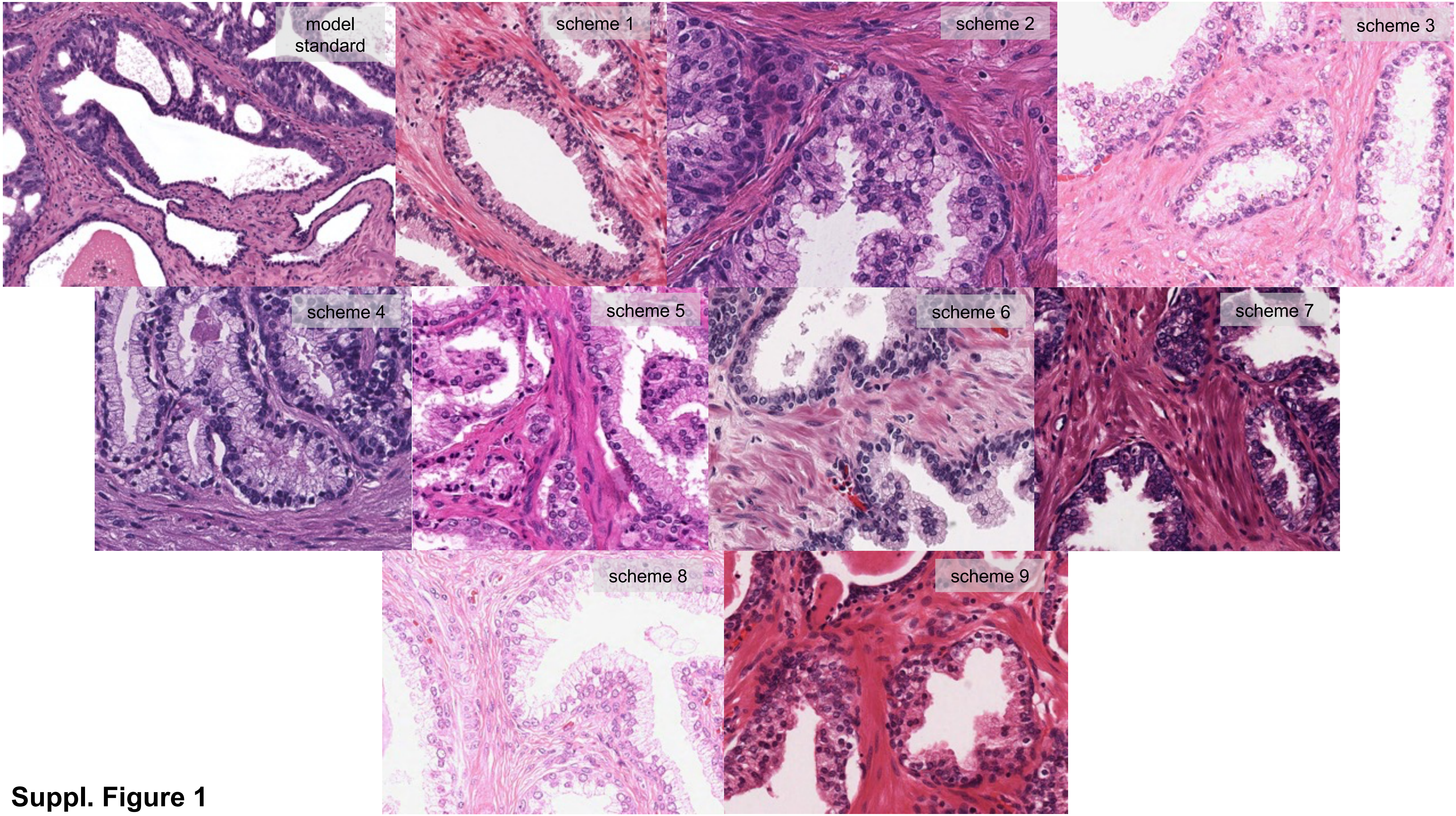

Suppl. Figure 1

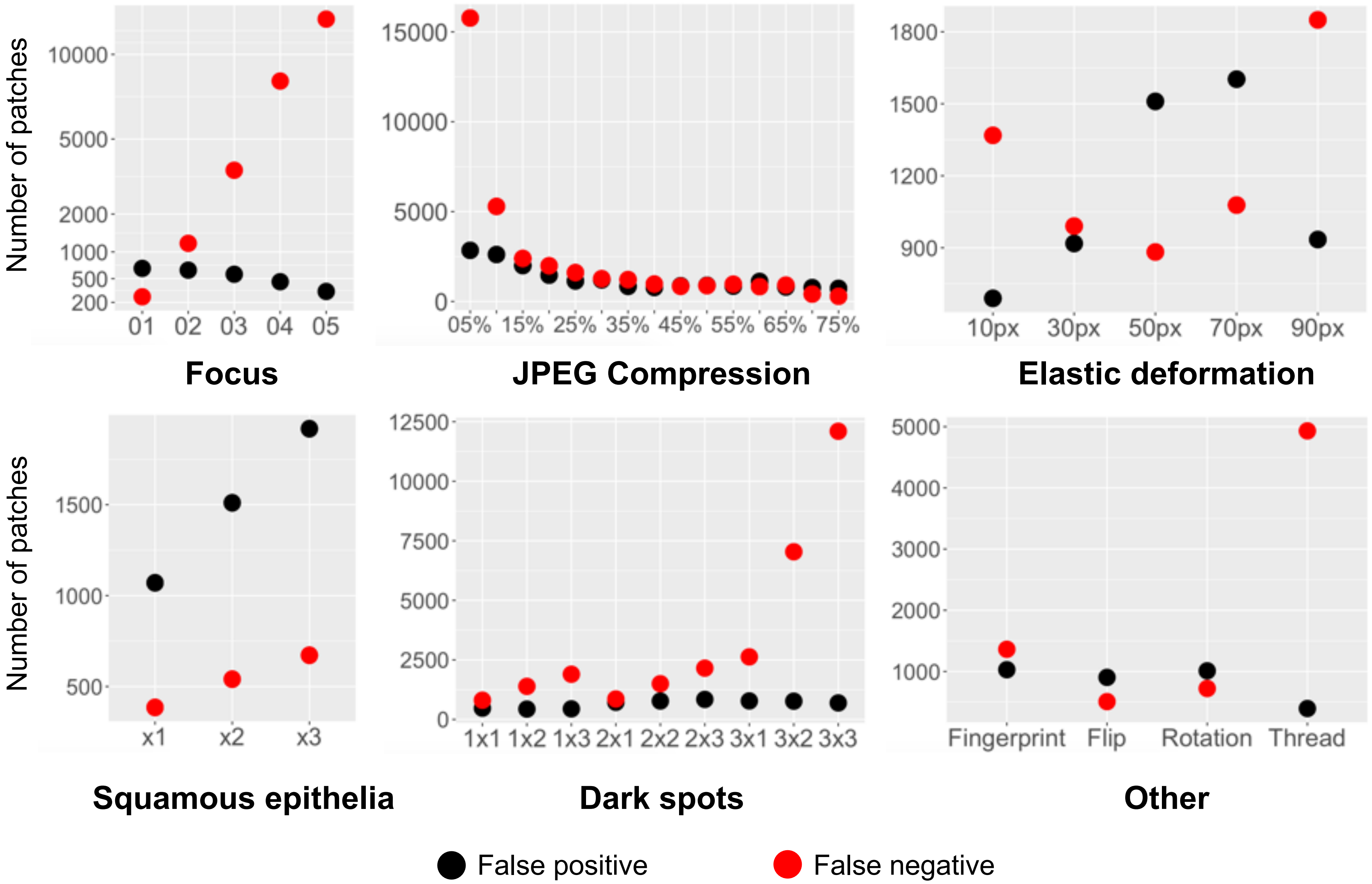

Patches, n	100	500	1000	2000	5000	10000
%FP	0.14%	0.7%	1.4%	2.8%	7.0%	14.0%
%FN	0.2%	1.0%	2.0%	4.0%	10.0%	20.0%

Suppl. Figure 2

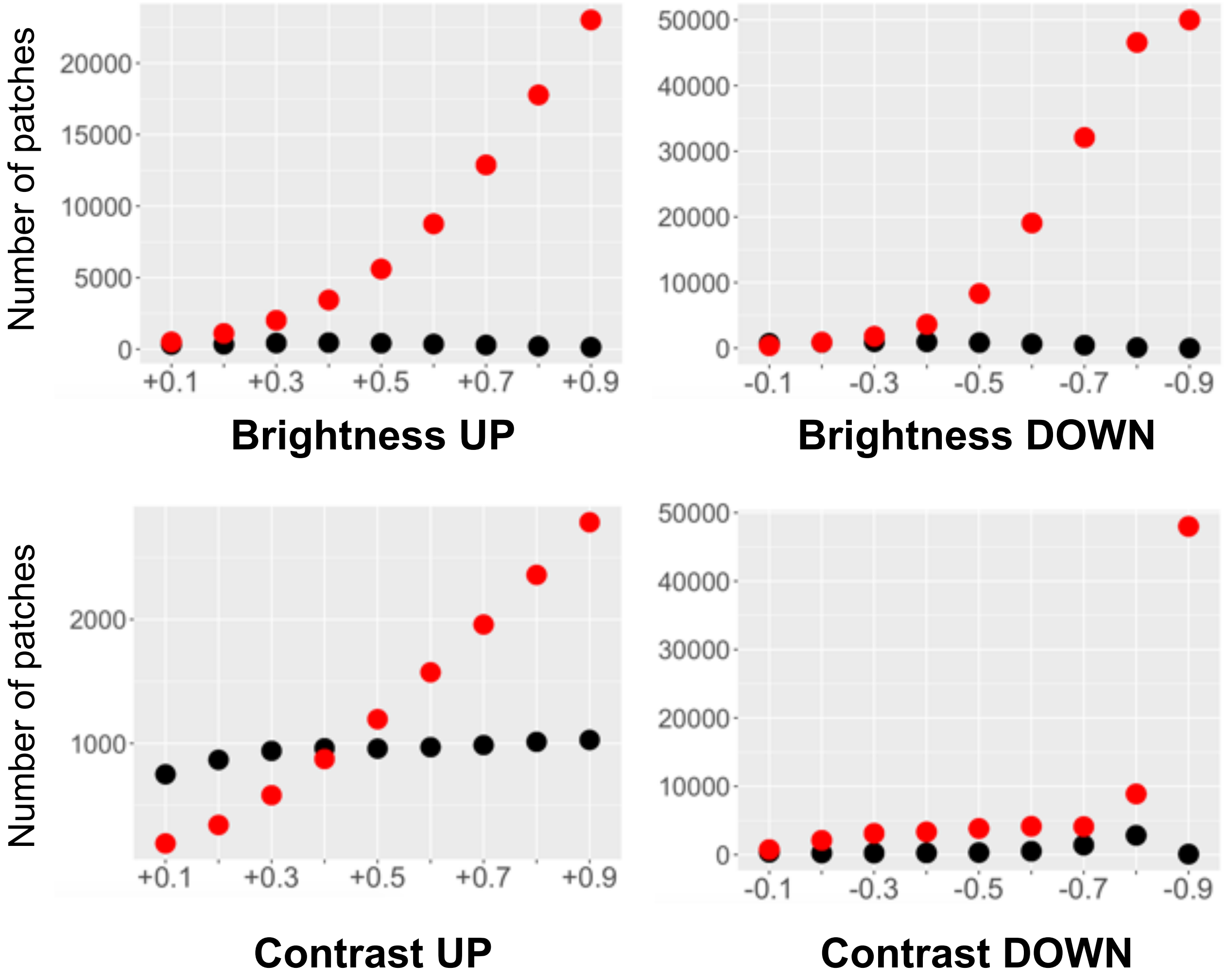

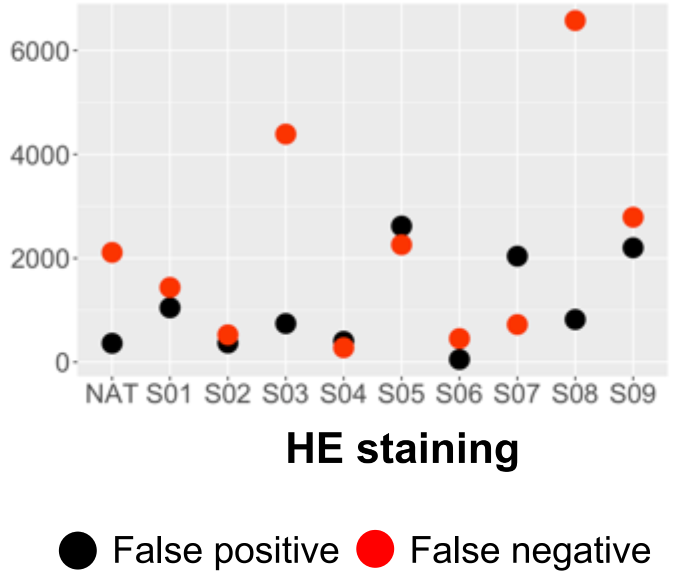

Patches, n	100	500	1000	2000	5000	10000
%FP	0.14%	0.7%	1.4%	2.8%	7.0%	14.0%
%FN	0.2%	1.0%	2.0%	4.0%	10.0%	20.0%

Suppl. Figure 3

Misclassified patches, false-positive

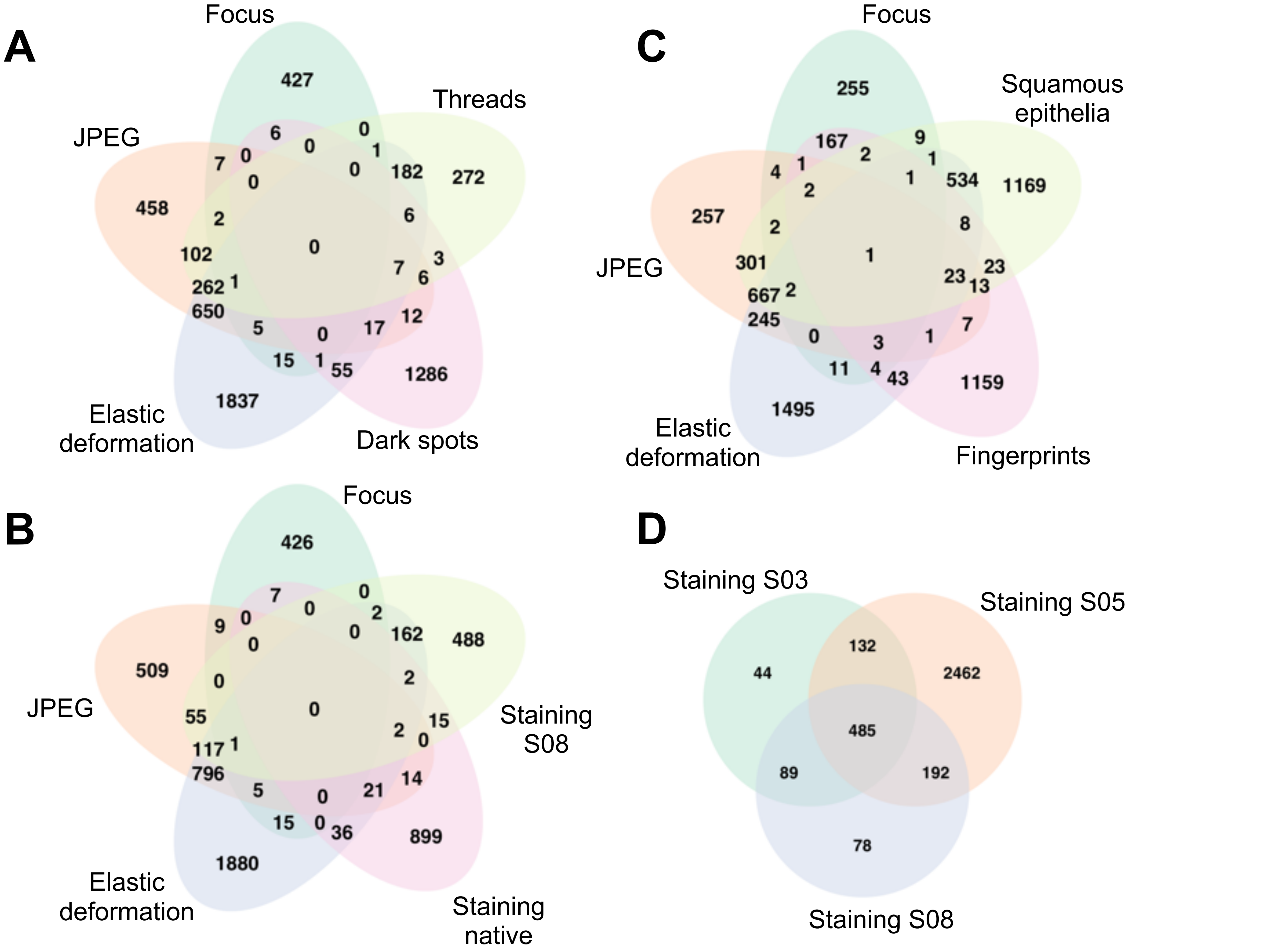

Suppl. Figure 4

Native staining | **Macenko stain transfer** | **Style transfer with pix2pix GAN**

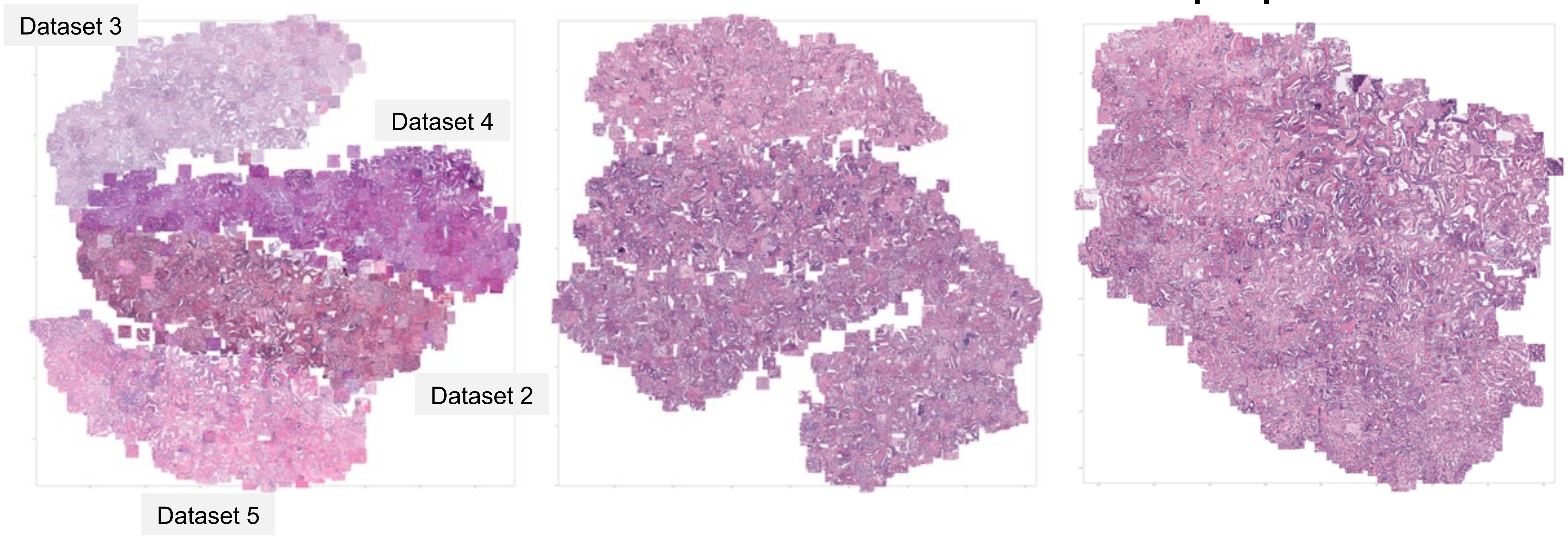

Suppl. Figure 5
